# Durable Response to Nivolumab Combined With Metformin in Advanced Pancreatic Cancer: A Case Report With Seven Years of Follow-Up

**DOI:** 10.7759/cureus.79001

**Published:** 2025-02-14

**Authors:** Ryosuke Sato, Katsuyuki Hotta, Toshio Kubo, Shigeru Horiguchi, Hironari Kato, Kazuyuki Matsumoto, Toshiyuki Kozuki, Heiichiro Udono, Katsuyuki Kiura, Motoyuki Otsuka

**Affiliations:** 1 Department of Gastroenterology and Hepatology, Okayama University Hospital, Okayama, JPN; 2 Center for Innovative Clinical Medicine, Okayama University Hospital, Okayama, JPN; 3 Department of Allergy and Respiratory Medicine, Okayama University Hospital, Okayama, JPN; 4 Department of Gastroenterology, Okayama City Hospital, Okayama, JPN; 5 Department of Respiratory Medicine and Allergology, Kochi Medical School, Kochi University, Kochi, JPN; 6 Department of Immunology, Okayama University Graduate School of Medicine, Dentistry and Pharmaceutical Sciences, Okayama, JPN

**Keywords:** immune checkpoint inhibitors, immunotherapy, metformin, nivolumab, pancreatic cancer

## Abstract

We report a case of poorly differentiated pancreatic cancer that showed an exceptional response to combination therapy with nivolumab and metformin. A 58-year-old man presented with epigastric pain and was diagnosed with locally advanced pancreatic cancer with para-aortic lymph node metastasis. After disease progression following modified FOLFIRINOX therapy (a combination of fluorouracil, leucovorin, irinotecan, and oxaliplatin), the patient was enrolled in a phase Ib clinical trial of nivolumab (3 mg/kg biweekly) combined with metformin (750 mg/day). Post-treatment imaging showed marked tumor shrinkage with normalization of the tumor markers. During treatment, the patient was diagnosed with early-stage lung cancer and underwent successful left S1+S2 segmentectomy with temporary suspension of immunotherapy. The therapeutic response of pancreatic cancer has been sustained for seven years, with minimal residual disease. This unprecedented response duration is particularly noteworthy considering his microsatellite stability, which typically predicts a limited response to immune checkpoint inhibition.

This case demonstrates an exceptional response to nivolumab and metformin combination therapy in poorly differentiated pancreatic cancer. The remarkable durability of the response suggests the need for further investigation to identify patients most likely to benefit from this therapeutic approach.

## Introduction

Pancreatic ductal adenocarcinoma (PDAC) remains a formidable therapeutic challenge, with projections indicating that it will become the second leading cause of cancer-related death by 2030 [1]. Despite advances in systemic therapies, the five-year survival rate remains approximately 10% [1]. Two standard first-line chemotherapy regimens have demonstrated survival benefits in metastatic pancreatic cancer: modified FOLFIRINOX (mFFX; a combination of fluorouracil, leucovorin, irinotecan, and oxaliplatin), and gemcitabine plus nab-paclitaxel (GnP). The PRODIGE 4/ACCORD 11 trial showed that mFFX improved overall survival relative to gemcitabine monotherapy [2] while the MPACT trial demonstrated a survival benefit with GnP over gemcitabine alone [3].

Monotherapy with immune checkpoint inhibitors has shown limited efficacy in patients with pancreatic cancer. In early clinical trials, the objective response rate was only 0-3% in unselected patients [4]. More recent studies combining checkpoint inhibitors with chemotherapy have shown modest improvements but still fall short of the desired outcomes [5]. However, metformin, beyond its established role as a first-line medication for type 2 diabetes mellitus, may exhibit significant antitumor effects through multiple mechanisms, including modulation of the adenosine monophosphate-activated protein kinase (AMPK)/mechanistic target of the rapamycin pathway [6]. Additionally, recent studies have suggested synergistic effects between metformin and immune checkpoint inhibitors through multiple mechanisms. Metformin may enhance anti-tumor immunity by reducing immunosuppressive cells in the tumor microenvironment and promoting T-cell function through metabolic modulation [7].

We report a case of microsatellite stable pancreatic cancer that showed an unprecedented durable response to combination therapy with nivolumab and metformin for more than seven years.

## Case presentation

A 58-year-old man presented at our institution with progressive epigastric pain. He had a history of hypertension treated with amlodipine. A physical examination revealed mild tenderness in the epigastric region, without rebound or guarding. No jaundice, hepatosplenomegaly, or palpable abdominal masses were observed. The patient's vital signs were stable. Initial laboratory tests demonstrated that the patient’s complete blood count, liver function tests, and renal function tests were within the normal ranges. However, the carcinoembryonic antigen (CEA) level (93.77 ng/mL (reference range: 0-5.0 ng/mL)) was elevated, while the carbohydrate antigen (CA) 19-9 level (12.2 U/mL (reference range: 0-35.4 U/mL)), the duke pancreatic monoclonal antigen type 2 (DUPAN-2) level (53 U/mL (reference range: 0-150 U/mL)), and the serum s-pancreas-1 antigen (SPan-1) level (12.9 U/mL (reference range: 0-30 U/mL)) were within the normal ranges.

**Figure 1:**
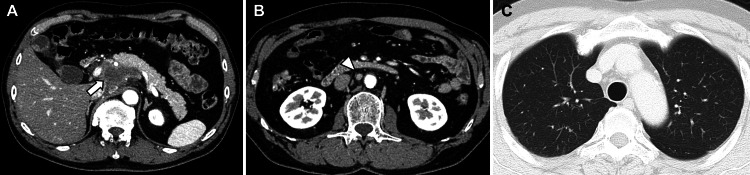
Imaging findings on initial examination (A) Contrast-enhanced computed tomography (CT) revealed a 60-mm hypovascular pancreatic head tumor (arrow) invading the common hepatic artery, proper hepatic artery, and portal vein. (B) A para-aortic lymph node was enlarged to 10 mm, suggesting metastasis (arrowhead). (C) No lung lesion was observed at first.

Contrast-enhanced computed tomography (CT) revealed a 60-mm hypovascular pancreatic head tumor with invasion into the common hepatic artery, proper hepatic artery, and portal vein, consistent with unresectable locally advanced pancreatic cancer (Figure 1A). The tumor had irregular margins with heterogeneous enhancement and areas of low attenuation, suggesting necrosis. Additionally, the para-aortic lymph node was enlarged to 10 mm in diameter (Figure 1B). No other distant metastases were identified and there were no obvious lung nodules (Figure 1C).

**Figure 2:**
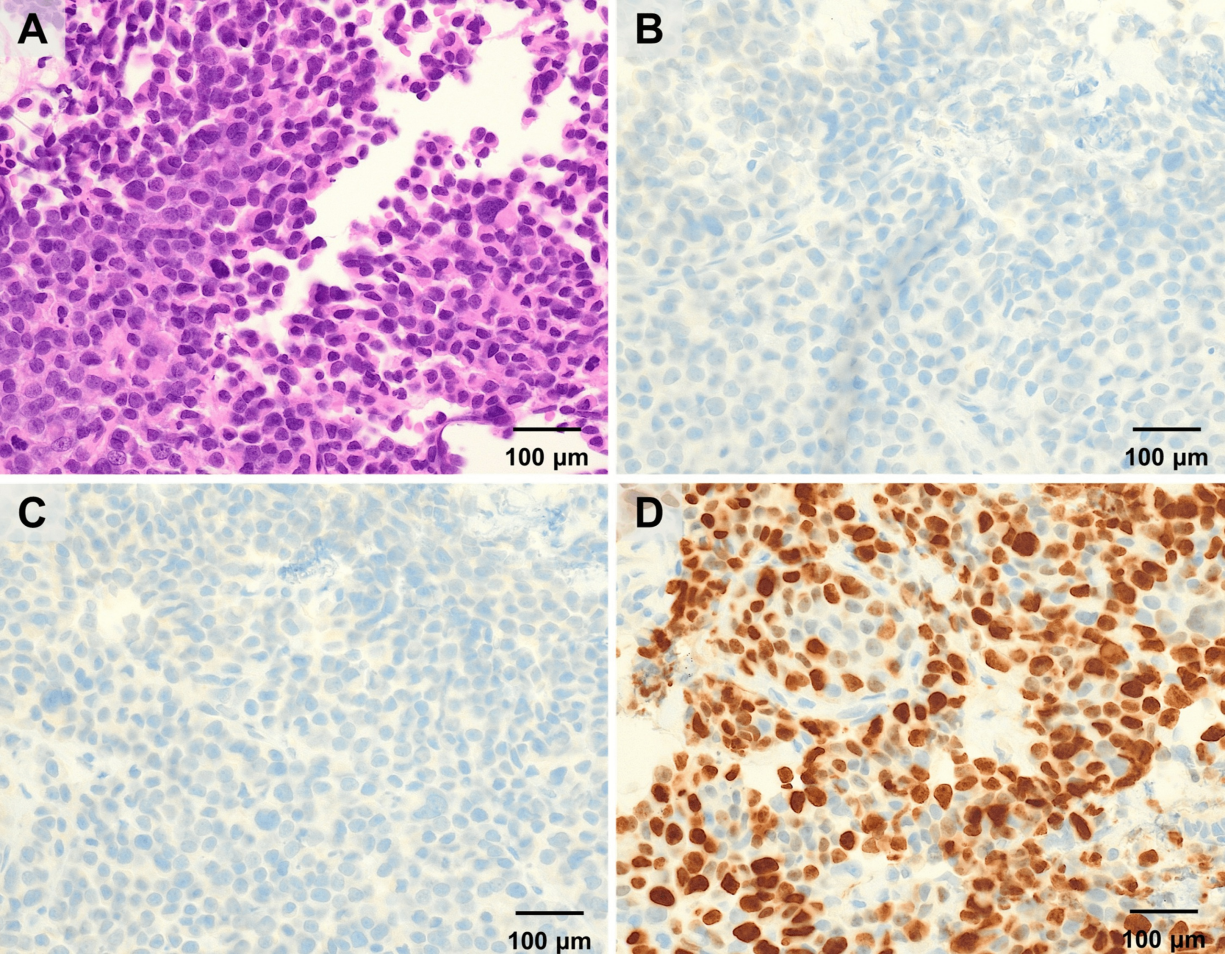
Pathological findings (A) The pancreatic tumor tissue collected by ultrasonography-guided fine-needle aspiration (EUS-FNA) demonstrated poorly differentiated adenocarcinoma with increased chromatin forming solid nests with invasive growth patterns on hematoxylin-eosin staining. (B) Immunohistochemical staining was negative for chromogranin A. (C) Synaptophysin was also negative. (D) The Ki-67 index was > 70%.

Endoscopic ultrasonography-guided fine-needle aspiration (EUS-FNA) was performed, and histological examination revealed poorly differentiated adenocarcinoma with increased chromatin-forming solid nests and invasive growth patterns (Figure 2A). The possibility of a pancreatic neuroendocrine neoplasm was also considered; however, immunohistochemical staining was negative for chromogranin A (Figure 2B) and synaptophysin (Figure 2C), with a Ki-67 index of > 70% (Figure 2D), confirming the diagnosis of poorly differentiated pancreatic ductal adenocarcinoma. Microsatellite stability testing (FALCO Biosystems Ltd., Kyoto, Japan) revealed a microsatellite stable (MSS) status.

**Figure 3:**
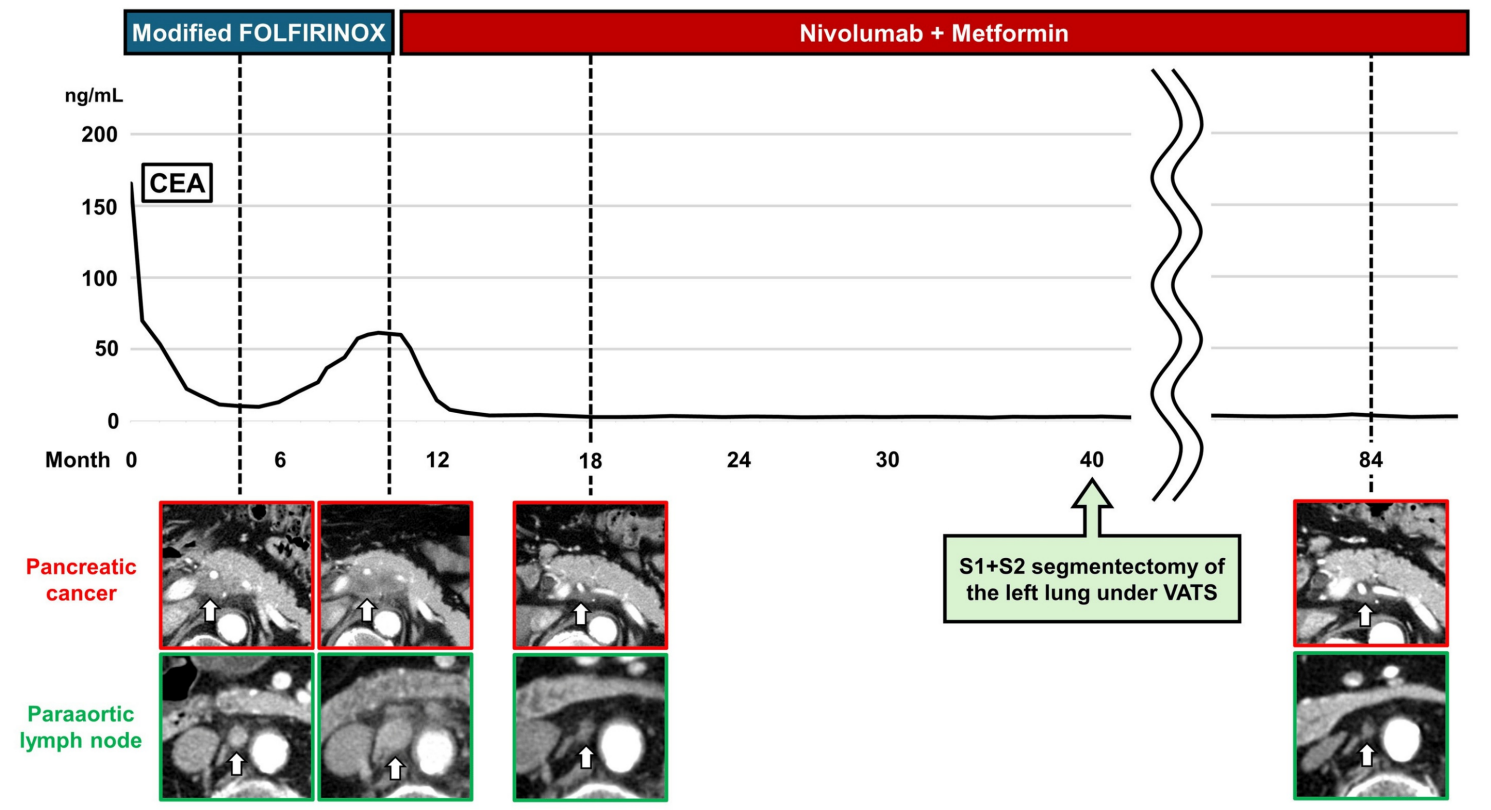
Clinical course and treatment response The initial computed tomography (CT) scan revealed locally advanced pancreatic cancer with paraaortic lymph node metastasis. After disease progression on modified FOLFIRINOX (fluorouracil, leucovorin, irinotecan, and oxaliplatin), nivolumab combined with metformin therapy was initiated, achieving marked tumor shrinkage sustained for seven years. The graph shows the changes in tumor markers (carcinoembryonic antigen; CEA) corresponding to the treatment response. CT images demonstrate a dramatic reduction in the size of the primary tumor and metastatic lymph node (arrow).

The patient received modified FOLFIRINOX therapy (oxaliplatin (85 mg/m²), irinotecan (150 mg/m²), leucovorin (200 mg/m²), and 5-fluorouracil (2400 mg/m²) continuous infusion over 46 h, every 2 weeks). The initial response assessment after 8 cycles showed a partial response according to the Response Evaluation Criteria in Solid Tumors (RECIST) version 1.1, with a 35% reduction in tumor size. However, after 10 months of treatment, CT demonstrated disease progression, with regrowth of pancreatic cancer to 43 mm and growth of the metastatic para-aortic lymph node to 12 mm, accompanied by CEA elevation to 61.50 ng/mL (Figure 3).

Because he did not want to undergo second-line treatment with gemcitabine plus nab-paclitaxel due to concerns about adverse events, he was subsequently enrolled in a clinical trial of nivolumab combined with metformin therapy at Okayama University Hospital [8]. Prior to starting this treatment, laboratory tests showed the following: white blood cell count, 5640/μL (reference range, 3300-8600/μL); hemoglobin, 13.6 g/dL (reference range, 14.0-18.0 g/dL); platelet count, 19.2×104/μL (reference range, 15.8-34.8×104/μL); total bilirubin, 0.94 mg/dL (reference range, 0.40-1.50 mg/dL); aspartate transaminase, 17 IU/L (reference range, 13-30 IU/L); alanine transaminase, 13 IU/L (reference range, 10-42 IU/L); creatinine, 0.91 mg/dL (reference range, 0.65-1.07 mg/dL); and HbA1c, 5.0%. The Eastern Cooperative Oncology Group (ECOG) performance status score was 1.

Treatment was initiated with metformin (250 mg, three times daily (total 750 mg/day)) and nivolumab (3 mg/kg, biweekly). The tumor response was evaluated every eight weeks using the RECIST (Response Evaluation Criteria in Solid Tumours) criteria. Remarkably, the first post-treatment images showed marked tumor shrinkage, and the levels of tumor markers, including CEA, normalized (Figure 3). The treatment was well-tolerated, with no adverse events exceeding Grade 1.

**Figure 4:**
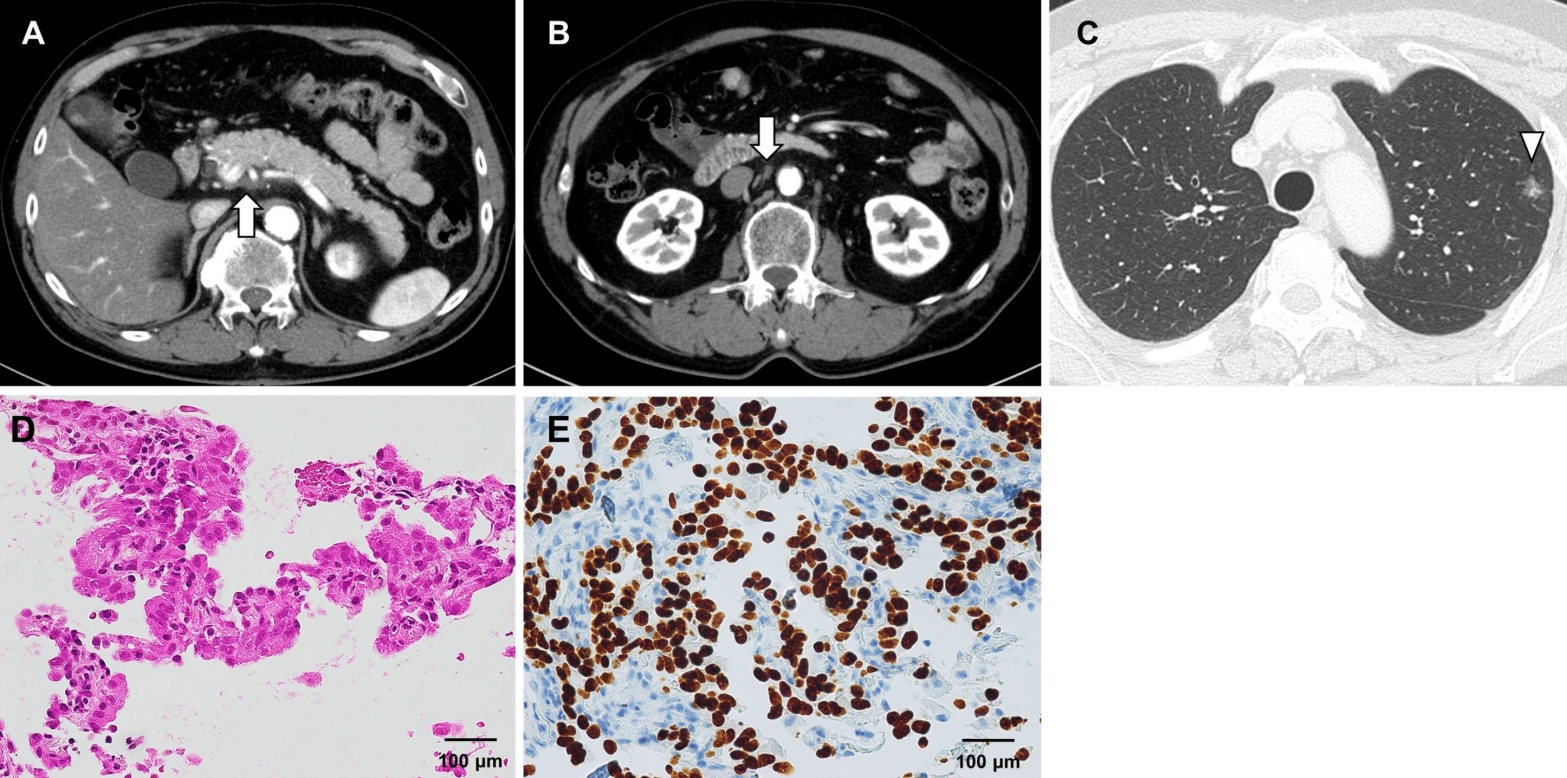
CT findings at 40 months (A, B) The diameter of the pancreatic cancer and para-aortic lymph node (arrow) has been continuing to shrink. (C) An 11-mm ground-glass opacity was observed in the upper lobe of the left lung (arrowhead). (D, E) A CT-guided lung tumor biopsy revealed the presence of thyroid transcription factor-1 (TTF-1)-positive adenocarcinoma with alveolar epithelial replacement.

After 40 months of therapy, routine surveillance CT revealed an enlarged left lung lesion, which was subsequently confirmed to be primary lung adenocarcinoma (T1miN0) by a CT-guided biopsy, which revealed thyroid transcription factor-1 (TTF-1)-positive adenocarcinoma (Figure 4). Following successful left S1+S2 segmentectomy under video-assisted thoracic surgery, immunotherapy was temporarily suspended for two weeks perioperatively and then resumed without complications. No postoperative complications were observed, and the patient recovered fully within two weeks.

The therapeutic response of pancreatic cancer has been sustained for seven years, with follow-up imaging showing a minimal residual disease in the pancreatic body (size, 17 mm) and a marked reduction in the size of the metastatic para-aortic lymph node (size, 5 mm) (Figure 3). The patient has maintained an excellent quality of life with an ECOG performance status of 0 and has continued to work full-time. He currently continues to receive the same dose of nivolumab and metformin, with regular monitoring every three weeks.

## Discussion

This case demonstrates an exceptional therapeutic response to nivolumab and metformin combination therapy in MSS pancreatic cancer, challenging our current understanding of immunotherapy resistance in this malignancy. The complete response that has been sustained for over seven years stands in marked contrast to historical data [4], where immune checkpoint inhibitor monotherapy yields response rates of only 0-3%. These findings have been further corroborated in phase I studies of single-agent anti-programmed cell death-1 (PD-1) therapy [9]. Even in more recent trials combining checkpoint inhibitors with chemotherapy, response rates rarely exceed 20%, and the duration of response is typically measured in months rather than years [10].

The significance of this durable response is even more noteworthy given the MSS status of the tumor. MSS tumors typically demonstrate a limited response to immune checkpoint inhibition alone [11], with most studies showing minimal clinical benefit in this molecular subtype. For example, dual checkpoint blockade with durvalumab and tremelimumab has demonstrated limited efficacy in MSS pancreatic cancer, with objective response rates of only 3.1% and a median progression-free survival time of approximately 2 months [5].

The unprecedented response in this case may be attributed primarily to the high programmed cell death-ligand 1 (PD-L1) expression characteristic of poorly differentiated pancreatic cancers. In this case, while PD-L1 immunostaining could not be performed as the EUS-FNA specimen was fully utilized for histopathological diagnosis and microsatellite stability testing, the exceptional response despite MSS status strongly suggests that this tumor likely had high PD-L1 expression. Recent studies have demonstrated PD-L1 positivity rates of up to 65-77% in this specific subtype [12,13], suggesting increased susceptibility to PD-1/PD-L1 blockade. This biological feature likely provided the foundation for effective immunotherapy in our patient, and favorable clinical factors, including the relatively good performance status at treatment initiation and the absence of a heavy tumor burden, may also be involved.

In addition, the combination therapy with metformin appears to fundamentally alter the therapeutic landscape. The contribution of metformin is twofold. Primarily, it exerts direct antineoplastic effects through inhibition of the AMPK-mediated mechanistic target of rapamycin complex 1 [14], particularly in pancreatic cancer, where metabolic reprogramming drives disease progression [15]. Second, and perhaps more crucially, metformin reshapes the tumor microenvironment [16]. By reducing immunosuppressive M2 macrophages while promoting the dendritic cell function, metformin may convert an immunologically "cold" tumor into one that is amenable to checkpoint inhibition.

The metabolic modulation induced by metformin extends beyond direct tumor effects. By altering cellular energetics and glucose utilization [17], metformin may simultaneously create an unfavorable environment for tumor growth while enhancing immune cell functions through the alteration of cellular energetics and glucose utilization. Recent studies have demonstrated that this dual metabolic-immunologic activity could be particularly significant in pancreatic cancer, where dense stroma and metabolic derangement typically limit the efficacy of immunotherapy [7].

The durability of the response observed in this case, particularly following treatment interruption for intercurrent lung cancer, suggests the establishment of effective immunological memory. This finding has important implications for the duration of treatment and the potential for treatment discontinuation in selected patients who achieve deep responses.

Several critical questions have emerged from this case. First, the identification of predictive biomarkers is paramount for patient selection. Second, the optimal duration of therapy for patients who respond to treatment requires clarification. Third, understanding the resistance mechanisms could inform strategies to expand the benefits of this combination approach.

This case provides a compelling rationale for the rigorous investigation of metformin-immunotherapy combinations in pancreatic cancer, particularly in molecularly defined patient subsets. The remarkable durability of the response, coupled with the favorable toxicity profile, suggests that this approach may represent a significant advancement in treating this historically refractory malignancy.

## Conclusions

This case demonstrates an unprecedented durable response to nivolumab and metformin combination therapy in a patient with microsatellite-stable pancreatic cancer. The seven-year sustained response, particularly noteworthy in light of the patient's MSS status, which typically predicts a poor response to immunotherapy, represents a significant departure from historical outcomes in pancreatic cancer treatment.

The remarkable efficacy observed in this case of poorly differentiated pancreatic cancer, which is typically characterized by the high expression of PD-L1, suggests that the combination of nivolumab and metformin may offer a promising therapeutic strategy for specific molecular subtypes of pancreatic cancer.
