# Correction: Durable Response to Nivolumab Combined With Metformin in Advanced Pancreatic Cancer: A Case Report With Seven Years of Follow-Up

**DOI:** 10.7759/cureus.c365

**Published:** 2025-10-16

**Authors:** Ryosuke Sato, Katsuyuki Hotta, Toshio Kubo, Shigeru Horiguchi, Hironari Kato, Kazuyuki Matsumoto, Toshiyuki Kozuki, Heiichiro Udono, Katsuyuki Kiura, Motoyuki Otsuka

**Affiliations:** 1 Department of Gastroenterology and Hepatology, Okayama University Hospital, Okayama, JPN; 2 Center for Innovative Clinical Medicine, Okayama University Hospital, Okayama, JPN; 3 Department of Allergy and Respiratory Medicine, Okayama University Hospital, Okayama, JPN; 4 Department of Gastroenterology, Okayama City Hospital, Okayama, JPN; 5 Department of Respiratory Medicine and Allergology, Kochi Medical School, Kochi University, Kochi, JPN; 6 Department of Immunology, Okayama University Graduate School of Medicine, Dentistry and Pharmaceutical Sciences, Okayama, JPN

This article has been corrected to include previously omitted conflicts of interest:

**1) Financial Relationships:** The statement "All authors have declared that no financial support was received from any organization for the submitted work" has been corrected as follows: "The research funding and investigational agent (nivolumab) were provided by Ono Pharmaceutical Co., Ltd."

**2) Payment/services info:** The statement "All authors have declared that they have no financial relationships at present or within the previous three years with any organizations that might have an interest in the submitted work." has been corrected as follows: 

"Katsuyuki Hotta reports honoraria from AZ, Chugai, Lilly, MSD, BMS, Ono, Boehringer-Ingelheim, Nippon Kayaku, Amgen, Taiho, Merck, Janssen, and Takeda, and grants from MSD, AZ, Lilly, BMS, Abbvie, and Janssen, outside the submitted work. 

Toshio Kubo reports honoraria from Chugai outside the submitted work. 

Toshiyuki Kozuki reports honoraria from from Chugai, AZ, Lilly, Taiho, BMS, Ono, MSD, Pfizer, Nippon Beohringer Ingelheim, Merck Biophama, Nippon Kayaku, Novartis, Daiichi-Sankyo, Takeda Pharmaceutical, Bayer, AMGEN, Janssen, Eisai, Kyorin, and Otsuka Pharmaceutical, and grants from AZ, Chugai, Lilly, Taiho, BMS, Ono, MSD, Kyowa Hakko Kirin, Merck Biopharma, Daiichi-Sankyo, AMGEN, Abbvie, Sanofi, Eisai, Labcorp Development Japan, IQVIA Services Japan, Giliead Sciences, Pfizer, and Bayer, outside the submitted work. He also reports consulting fees from Chugai, AZ, Ono, Pfizer, Daiichi-Sankyo, Bayer, Abbvie, Nippon Beohringer Ingelheim, Regeneron, and GSK, outside the submitted work.

Other authors have declared that they have no financial relationships at present or within the previous three years with any organizations that might have an interest in the submitted work."

